# Stressful Life Events and Chinese Older People Depression: Moderating Role of Social Support

**DOI:** 10.3389/fpubh.2021.768723

**Published:** 2021-12-17

**Authors:** Xiao Yu, Shu Liu

**Affiliations:** ^1^Northeast Asian Research Center, Jilin University, Changchun, China; ^2^Northeast Asian Studies College, Jilin University, Changchun, China

**Keywords:** stressful life event, depression, Chinese older people, social support, family support, social activity

## Abstract

**Aim:** This study analyzes the effects of retrospective stressful life events on current depression among Chinese older people and how these effects are moderated by social support. Stressful life events comprise bereavement, divorce, health adversities, accidents, and financial losses due to fraud.

**Data and Method:** Data were drawn from the China Health and Retirement Longitudinal Study (CHARLS) of the 2015 panel, and responses from 9,619 older people aged over 60 years were used. The least-squares regression method was applied to measure the linear effects. Propensity score matching minimized selection bias and enabled the measurement of the net effects of stressful life events. The bias-corrected matching estimator was also used to correct the inexact matching bias from propensity score matching.

**Result:** Experienced stressful life events and exposure to cumulative stressful life events were found to lead to depression in older people. When older people experienced stressful life events but with more social activities, and higher satisfaction and frequent contact with children, their depressive levels were lower. The results of the propensity score matching showed that stressful life events resulted in depression in older people. Furthermore, individuals with family support were able to moderate stressful life events effects; however, the moderating effects of social activity separately were negligible. In sum, with the moderating role of family support and social activity, the average differences in older people depression caused by stressful life events decreased.

**Conclusion:** Experiencing stressful life events is detrimental to the psychological health of the older people. Social support, including family support and social activity, has buffered detrimental effects on depression caused by stressful life events.

**Interpretations:** The study underscores the need to supply effective interventions for the older people who experienced stressful life events. First, society should improve the capability of community care centers to supply mental health services. Second, family members should pay attention to mental condition of older people, and specific support should concord with the needs of Chinese older people. In addition, support suppliers can move from being confined to kinship relationships to close relationships, such as the community partners and neighbors.

## Introduction

Mental illness is a significant public health problem. In 2015, 4.4% of the global population was diagnosed with depression, over an estimated 300 million people (1). In recent years, the prevalence of mood and anxiety disorders in China has been on the rise, with a point prevalence of 1.1% in both 1982 and 1993, a 12-month prevalence of 7.0% in 2002, and a lifetime prevalence of 1.3% in 1982, 1.4% in 1993, and 13.2% in 2002 (2). The weighted prevalence of any mental disorder excluding dementia was reported to be 9.3% for the preceding 12 months and 16.6% for lifetime prevalence; the prevalence of depression was 4.2% in 2017, as estimated by the World Health Organization (1, 2). Depression is a long-term and easily recurrent mental disorder that can cause considerable loss in health and functioning, including functional impairment, low quality of life, high risk of suicide, and immense burdens not only for individuals but also for families and societies (1, 3–6). Depression is ranked as the largest single factor of disability worldwide (1). Furthermore, there is a huge imbalance between mental disorder burdens—which account for 12% of the global burden of disease—and mental health expenditures, which account for <1% of health expenditures in most countries (7, 8). The World Health Organization (1) quantifies the health losses from mental disorders in terms of years lived with disability, which is the product of the prevalence of mental disorders and the average level of disability related to them. Chinese depressive disorders contributed 7.3% of the total years lived with disability as a health loss.

Depression in old age is common; indeed, the prevalence of depression peaks between 55 and 74 years of age (1, 5, 9). According to the Chinese National Censuses, the proportion of the population aged 60 and over was 10.45% in 2000, 13.31% in 2010, and 18.70% in 2020, and that aged 65 and over was 7.09% in 2000, 8.91% in 2010, and 13.50% in 2020 (10–12). These proportions have been increasing gradually with the increase in the Chinese population.

Stressful life events are risk factors for depression (1, 13). 70% of respondents report experiencing at least one traumatic event in their life course, and the majority have been exposed to multiple events (14). Old age is a period of constant loss: Various risks, such as disease, widowhood, and departure of loved ones, are difficult to avoid in old age (15, 16). Therefore, older people, as high-risk groups, are exposed to the prevalence of depression and experience stressful life events.

The perceived social support to the older people can influence the possibility of their depression (17, 18). As a country influenced by Confucian culture, the tradition of filial piety and respect for older people in the Chinese culture encourages family support to play a certain role to moderate the effects of depression resulting from stressful life events.

Currently, research is focused on Chinese older people, but few studies have investigated depression in Chinese older people (5, 15, 19, 20). These studies were mostly based on medical theories describing current conditions and risk factors for depression. Furthermore, some societal studies explore effects on depression from social support and stressful life events separately. Through this process, social support has covered diversified interpretations and its referral is ambiguous. Some studies use social support to refer to social activities, where social support and social activities are overlapped in contents and are interchangeable (21–23). Otherwise, social support has narrowed its definition to family support, used as a factor similar with social activity to discuss health problems (24–26).

Therefore, this study mainly discusses two aspects: first, the relationship between stressful life events experienced by the Chinese older people and depression, and second, how social support moderates the effects of stressful life events on depression in older people. Depression was measured in terms of depressive feelings and behaviors rather than clinical diagnoses. Furthermore, the study first describes social support broadly in this paper as interactions from certain contexts, either in the society or community or in the family to enhance individual's well-being; second, the study differentiates social support into two types based on the place of references, inside or outside of family: family support and social activities. The study makes use of the 2015 China Health and Retirement Longitudinal Study (CHARLS) panel data, applies multiple regression to detect the risk factors for depression, and carries out propensity score matching (PSM) to quantify, first, the effects on depression from stressful life events, and second, the buffering effects on depression from stressful life events via social activities, family support and social support, under the counterfactual framework.

## Literature Review

### Risk Factors for Depression

Age has been regarded as a high-risk factor for depression because events such as bereavement, illnesses, and disabilities are inevitable in old age (9, 15, 19, 27). In general, women are at a higher risk for depression than men. However, such gender differences decrease in old age (4–6, 20, 28, 29). Indeed, females are distressed by network relationships, but males are affected by job or financial problems, both of which lead to depression (19). Illnesses, functional impairment, and perceived deteriorating health are also highly correlated with depression (5, 6, 9, 19, 27, 28). Chronic illnesses, that leads to functional decline, can be regarded as a risk factor for depression (30). Further, low educational levels and low socioeconomic status have been shown to result in a high risk of depression (4, 5, 29).

Bereavement, including widowhood and loss of offspring, is consistently and strongly related to depression (28, 31–33). However, when facing widowhood, older women are less vulnerable than older men (34). Stressful life events are still risk factors for depression and are predictors of the onset and relapse of depression (5, 6, 15, 19, 28, 35–37). However, previous research has covered many risk factors related to depression but only partly underlined the association between stressful life events and depression (20).

### Stressful Life Events and Depression

Life stress is caused by adverse social-environmental experiences, which include economic circumstances, physical health, mental states, and social relationships (36). Stressful life events are defined as transitions that prompt a need to readjust the daily life routine, especially emotional and physical readjustment, and include events, such as death and dying, and issues related to healthcare, finances, and family (15, 35, 38). Furthermore, stressful life events for older people include social losses, illnesses, changes in social roles, and changes in daily life patterns (15). In previous studies, stressful and negative life events have been treated as similar and interchangeable (20, 35).

Stressful life events have a modest but significant relationship with depression, but whether this is a causal effect is unclear (20, 32, 37, 39). There is a stronger association between stressful life events and depression when the event is more severe or there are more events (32, 39). Social readjustment rating scale measures the required social life adjustments associated with various life events, including some undesirable ones that are regarded as stressors (40). Research based on the social readjustment rating scale has shown a significant association between exposure to stressful life events and stress-related symptoms (37, 38, 41). However, in some studies, stressful life events have been shown to be causally associated with depression, and even early exposure to life stress can predict depression in later life (39, 42, 43). Furthermore, the causal association between stressful life events and depression is not unilateral: genetic risk factors and personality traits both result in stressful life events and depression (43).

However, most studies collectively consider effects from stressful life events on mental health, and do not differentiate the effects from cumulative stressful life events, which are a quantity of stressful life events older people once experienced. Cumulative violence exposures positively associate with the risk for poor health, and each additional violence exposure may increase 38% of the risk of poor health (44). The total number of stressful life events is related to current depression and depression in later life, where an increase in stressful life events has decreased a health score (20, 45, 46). Indeed, lifetime cumulative adversity rather than discrete stressful life events has a more lasting effect on health (47). For example, individuals who have more cumulative adversities will experience more health problems later (14).

There are two steps of assessing whether a life event is a stressor: first, determining whether the event occurred in a particular time span; second, appraising the event level (48). Time span, either short term or long term, has been regarded as an essential measure between stressful life events and depression. Events that occurred more recently were associated more strongly with severe stress symptoms (5, 37). Stress diminishes when events occurred at a more distant point in time. In contrast, lifetime traumatic events have been shown to have long-term impacts on physical and mental health (14, 49, 50). Indeed, events occurring earlier are still associated with late-life depression (20). Adults continue to experience emotional pain from bereavement for decades after the event (51). In addition, some studies do not set time limits when discussing stressful life events and depression.

### Social Support and Depression

The World Health Organization regards social support as an essential factor in health; indeed, family members, friends, and communal members are the main suppliers of social support (33). Social support is a perception of specific help from network partners and has different effects on individuals, either to enhance positive effects and further increase their well-being or to reduce the negative impacts of depression (6, 26, 52). Furthermore, the perceived social support and received social support are distinguished, where the former describes the subjective feeling of being supported by the relationships and the latter focuses on actual support; indeed, perceived social support is commonly measured and more effective in buffering depression than received support (17).

Greater social support plays a protective role against depression, whereas less social support may be a risk factor for current depression or depression in later life (26, 53). Furthermore, social support can erase sex-specific effects on depression (26). The convoy model of social relations argues that individuals' interactions with essential people—defined as not only close relations but also those who have an effective impact on the individual—are stable and long-term; indeed, the accumulation of interactions can enhance individuals' spiritual health (31). Self-determination theory explains the transmission mechanism of social support, where extrinsic social contexts have transformed into intrinsic self-values and self-motivation and conversely facilitates the ability to cope with social contexts (54).

Family support is a robust protective factor against depression in the older people (33, 34). First, individuals prefer to have effective support rather than network size; the former includes matches between emotional demand and related support, and the quality of social relationships (17, 18). Older people's social networks are characteristically smaller but stronger in emotional closeness with network partners. Second, social-emotional selective theory argues that the perception of time influences individual motivation (55). Older people's perceived limited time left to live improves their selective preferences toward emotionally close social partners (26, 52, 56). People in the United States and European countries prioritize friend networks; however, people in Asian countries regard family networks as important (3, 17, 57). Much social support is drawn from kin, and with age, the effects of relatives are more essential than non-kin relationships (58). Therefore, when addressing depression in the Asian context, families should be included in the plan for support and interventions.

Family support from adult children can improve older people's life quality and strengthen their life satisfaction, especially for the positive effects on their mental health (59, 60). In particular, older parents who received financial support from their children had enhanced self-esteem and lower negative mental outcomes. In this aspect, older people show similarities across genders with regard to positive effects from financial support (33, 34). Furthermore, family support from relatives can significantly alleviate the death anxiety of older people, which supplies a useful resource to help older people to relieve negative emotions and contain better psychological condition (61). Finally, older adults with strong family support can deal with stressful issues more effectively than those without (16, 26, 52).

Social activity is highly associated with good self-rated health and lower depressive symptoms (62, 63). Indeed, although social participation occurrence decreases with age, the influence of social participation on health status will increase with age (62). Therefore, social activity is a key to healthy aging (21). Older people participating in social activities can decrease the risk of depressive symptoms; indeed, more frequent and more diverse participation can further reduce the risk (64). Further, diverse activities rather than single activity can be more protective against depressive symptoms (65).

### Stressful Life Events, Social Support, and Depression

Social support plays a protective role against depression and acts as a moderator between stressful life events and depression (20, 28, 35, 36). Through social support, individuals enhance their resilience to cope with stressful life events, and consequently, depression decreases (36). Indeed, the older people have different responses toward social support stress-buffering effects, with the oldest older people showing the greatest response (16).

Therefore, the study proposes the following hypotheses:

Hypothesis 1: Chinese older people who experience stressful life events will show an increase in depression.
Hypothesis 2: When Chinese older people experience stressful life events, social activities may buffer its effect on their depression.
Hypothesis 3: When Chinese older people experience stressful life events, family support may moderate its effect on their depression.
Hypothesis 4: When Chinese older people experience stressful life events, social support may lessen its effect on their depression.

## Data and Methodology

### Methodology

#### Multiple Regression Analysis

The study used multiple regression of the ordinary least square estimates to present the linear relationship between stressful life events and depression, and the moderating effects of social support on the relationship. Additionally, social support and its components, social activities and family support, were considered separately.

The multiple regression equations are:


(1) $$\begin{array}{l} \;Y_{1}=\alpha +\beta_{1}MX+\beta_{2}CX+\varepsilon \\ Y_{2}=\alpha +\beta_{1}MX+{\beta_{2}CX+\beta }_{3}SA \end{array}$$

(2) $$\begin{array}{l} \;+\beta_{4}MX\times SA+\varepsilon \\ Y_{3}=\alpha +\beta_{1}MX+\beta_{2}CX+\beta_{5}FS \end{array}$$

(3) $$\begin{array}{l} \;+\beta_{6}MX\times FS+\varepsilon \\ Y_{4}=\alpha +\beta_{1}MX+\beta_{2}CX+\beta_{3}SA+\beta_{4}MX\times SA+\beta_{5}FS \end{array}$$

(4) $$\begin{array}{l} +\beta_{6}MX\times FS+\varepsilon \end{array}$$

where Y is depression, and the subscripts from 1 to 4 represent separate models: stressful life event model, social activity model, family support model, and social support model. MX are main independent variables, CX are control variables, SA and FS are moderator variables of social activities and family support, and intersection variables between main independent variables and moderator variables are presented. α and β are coefficients; and ε is the error term.

#### Propensity Score Analysis

This study aims to discuss the association between stressful life events and depression and quantify the effects of stressful life events and social support on depression. The study's program evaluation is based on counterfactual inferences; the treatment group comprises older people who have experienced stressful life events, and those who have not, comprise the control group.

Whether older people experience stressful life events is the outcome of self-selection. First, stressful life events are sorted into independent events (e.g., bereavement) and dependent events (e.g., divorce), where dependent stressful events result from individuals' behaviors (42, 66). Second, age is a trigger for stressful life events (16), and explains the fact that older people are a high-risk cohort to the problems of health adversities and bereavement. However, seniors of different age groups present diversified risks; for example, the young-old and older-old are exposed differently. Therefore, the initial limits of the treatment and control groups are different, and selection biases exist.

The effects of stressful life events on depression are measured as follows:


(5) $$\begin{array}{l} y_{i}=\{\begin{array}{c} y_{1i}\;D_{i}=1\\ y_{0i}\;D_{i}=0 \end{array} \end{array}$$

where *D*_*i*_ is a dummy variable that takes 1 if the respondent has experienced a stressful life event, and 0 otherwise; and *y*_*i*_ is the outcome of interest that represents depression in this study, with *y*_1*i*_ denoting depression from stressful life events and *y*_0*i*_ denoting depression when the respondent did not experience stressful life events.

Because the treatment effect is a random variable, the average treatment effect (ATE) is measured as:


(6) $$\begin{array}{l} ATE\equiv E\left(y_{1i}-y_{0i}\right) \end{array}$$

Average Treatment Effect on the Treated (ATT) is measured as:


(7) $$\begin{array}{l} ATT\equiv E\left(y_{1i}-y_{0i}|D_{i}=1\right) \end{array}$$

Given the observed covariate vector, the propensity score is the conditional probability that an observation case is assigned to a particular intervention (67). Based on the selection of observables, the ignorability assumption and matching assumption are satisfied.

The study uses PSM to identify older people who have not experienced stressful life events with similar characteristics to those who have, to further compare two-cohort differences in depression. The differences in depression result from the effects of experienced stressful life events. Social support and its components, social activities and family support, have been included separately as characteristics in models. PSM is conducted here in 3 steps: first, finding covariates that cause an imbalance between the treatment and control groups by logistic regression; second, matching replacements in the common support region; and third, applying a data balancing check to appraise the match quality (67, 68). This study applied one-to-one neighbor, radius, and kernel matchings to measure the propensity score.

When considering covariates in detail, the equations are:


(8) $$\begin{array}{l} ATT_{1}\equiv E\left(y_{1i}-y_{0i}|D_{i}=1,\;x=CX\right) \end{array}$$

(9) $$\begin{array}{l} ATT_{2}\equiv E\left(y_{1i}-y_{0i}|D_{i}=1,x=CX+SA\right) \end{array}$$

(10) $$\begin{array}{l} ATT_{3}\equiv E\left(y_{1i}-y_{0i}|D_{i}=1,x=CX+FS\right) \end{array}$$

(11) $$\begin{array}{l} ATT_{4}\equiv E\left(y_{1i}-y_{0i}|D_{i}=1,x=CX+SA+FS\right) \end{array}$$

where ATT is average treatment effect on the treated, and the subscripts from 1 to 4 represent separate models: stressful life event model, social activity model, family support model, and social support model. Covariates include control variables (CX), moderator variables of social activities (SA) and family support (FS). Compared to stressful life event model, the change of differences in depression separately attributes to moderating effects from social activities, family support and social support.

PSM relies on a sufficient sample. However, when the number of matches increases, it increases the difficulty of precise matching between the treatment and control groups (67). Bias-corrected matching estimators proposed to solve the problems of conditional biases from simple matching estimators in inexact matching (68). Bias-corrected matching estimators use vector norms to measure the distance of every observable covariate, rather than a one-dimensional score, for the application of PSM. This study uses a bias-corrected matching estimator to further verify the effects of stressful life events on depression.

#### Robustness Check

For the main model, the study selected the total number of stressful life events experienced by older people to present a linear relationship with depression by means of multiple regression analysis. After PSM analysis, the study shrunk the sample to matched individuals and investigated whether experienced stressful life events can be used as the main independent variable to further check the robustness of the model

### Data

The study uses 2015 panel data from the China Health and Retirement Longitudinal Study (CHARLS). CHARLS aims to collect a high-quality nationally representative sample of Chinese residents aged 45 years and older to serve the needs of scientific research on the older people. CHARLS was approved by the Institutional Review Board at Peking University. The IRB approval number for the main household survey, including anthropometrics, is IRB00001052-11015. Each interviewee who agreed to participate in the survey signed a written informed consent. The baseline national-level wave of data collection was in 2011, followed by 5 national waves in 2011, 2013, 2014, 2015, and 2018 (69). In order to be closer to reality, the study tends to select a recent panel; however, due to the time when the data was obtained, the study finally employs 2015 panel data from CHARLS.

First, based on individual ID, the study merged 8 data subsets into one to allow for the prerequisite of data washing, which included data on demographic background, family information, family transfer, healthcare, health status, individual income, and work, retirement, and pension. There were 21,069 observations in the sample (11,044 females and 10,025 males). Second, the study discarded observations beyond the specified age limits. The aim of this study was to discuss the association between stressful life events and depression among the older people. Therefore, the study retained data from individuals who are at least 60 years old, producing a sample of 9,785 individuals. However, individuals do not experience stressful life events randomly, and a personal history of depression exacerbates lifetime adversity and is a risk factor for experiencing stressful life events and depression in later life (20, 42, 43). Therefore, this study discarded individuals with clinically diagnosed depression to avoid endogeneity problems. Finally, 9,619 interviewees were included in the final sample.

In this study, stressful life events are conceptualized as exposure to bereavement and divorce, health adversities, accidents, and defrauded financial losses. Events of bereavement of spouse, child, and relatives and severe accidents fulfill the criteria of a traumatic event as described by the American Psychiatric Association (70): “Directly experiencing the exposure to actual or threatened death, serious injury, or sexual violence; or witnessing, in person, the event(s) as it occurred to others; or learning that the traumatic event(s) occurred to a close family member or close friend; or experiencing repeated or extreme exposure to aversive details of the traumatic event(s)” (page 182). Events of divorce and defrauded financial losses fulfill the criteria of an adjustment disorder, where the stressor can be of any severity or type rather than that required by traumatic events (page 187).

Event selections should consider the independence of events, that is, whether events are the outcomes of problems (41). Health is discarded in the selection, in that health is reflected as an individual's wellbeing but not a specific event (20). However, it is possible to cover this in the selection if a health comparison is included. Furthermore, including health events as the main independent variables rather than control variables will show a strong association with depression (28). Indeed, poor self-rated health is highly associated with depression (5, 19). Events of health adversities in this study cover poor self-rated health, hospitalization in the previous year due to illness, and functional disability. Due to health and memory problems, when the older people have at least one of these six difficulties, are unable to function independently without help, and when such a situation has not been resolved within 6 months, the study regards the older people as those who experienced stressful life events of functional disability. These difficulties include using the toilet, including getting up and down; getting into or out of bed; eating, such as cutting up food; bathing or showering; dressing; and controlling urination and defecation.

### Measures

#### Dependent Variable

##### Depression

This is a cumulative variable that is measuring by adding up the scores for responses to the question of how the respondent felt and behaved during the preceding week. The responses were: (1) I was bothered by things that do not usually bother me; (2) I had trouble keeping my mind on what I was doing, (3) I felt depressed, (4) I felt everything I did was in vain, (5) I felt hopeful about the future, (6) I felt fearful, (7) My sleep was restless, (8) I was happy, (9) I felt lonely, and (10) I could not continue my life. Negative items are assigned a value of 1, for responses of “rarely” or “none of the time;” a value of 2 for “some or a little of the time;” a value of 3 for “occasionally” or “a moderate amount of the time;” and a value of 4 for “most or all of the time.” Responses for positive items are conversely assigned from 1 for “most or all of the time” to 4 for “rarely or none of the time.” The value of Cronbach's alpha for these 10 items is 0.7950, which denotes a good credential of scales (71). The responses were summed up to measure depression in the older people.

#### Main Independent Variables

##### Number of Stressful Life Events Experienced

This variable is added to stressful life events experienced by the older people. There are eight stressful life events: widowhood and divorce, bereavement of child, bereavement of relatives in the past 12 months, being financially defrauded, limited daily activities due to earlier accident, poor self-rated health, functional disabilities, and hospitalization due to illness in the past 12 months.

##### Experienced Stressful Life Events

This is assigned a value of 1 for individuals who have experienced at least one of the stressful life events mentioned above, and 0 otherwise.

#### Moderator Variable of Social Activities

##### Social Activities

Social activity is defined as being involved in communities and engaging in productive activities, which presents the association between the social activities of the older people and depression (7, 18, 72). This is a cumulative variable based on the social activities of the older people once done in the last month. Social activities include (1) interactions with friends; (2) playing Mahjong, chess, or cards, or going to a community club; (3) helping friends or neighbors who do not live with you; (4) going to a sport, social, or other kinds of club; (5) taking part in a community-related organization; (6) voluntary or charity work; (7) caring for a sick or disabled adult who does not live with you; (8) attending an educational or training course; (9) online stock investment; and (10) other social activities. The study intersects variable of social activities with the number of experienced stressful life events and whether they experienced stressful life events. These two intersection variables represent the buffering effects of social activity between stressful life events and depression.

#### Moderator Variable of Family Support

##### Living Children

The variable of living children is a cumulative variable that measures how many living children the older people have. The quality of life of older people was negatively associated with the number of children. The proportion of older people with many children who believe that their children are not filial is significantly higher than that of older people with fewer children (73). Furthermore, too many children could shirk their responsibilities when caring for their parents, leading to a free-rider problem (74). Therefore, the study set a quadratic term for the number of living children to test the linear relationship between the number of living children and depression in older people.

##### Satisfaction With Relationships With Children

Responses to the question “How satisfied are you with your relationship with your children?” were ranked on a 5-point scale, with 1 standing for completely satisfied, 5 not at all satisfied. Furthermore, this variable intersects with main independent variables to present the moderating effects between stressful life events and depression.

##### Financial Support by Children

The variable is a binary, measured by the response to “In the past year, did you or your spouse receive economic supports at least 500 yuan (including money support and in-kind support) from your children?” that takes a value of one for an affirmative response and 0 otherwise. Furthermore, two intersection variables were set.

##### Financial Support by Relatives

This variable is a binary response to “In the past year, did you or your spouse receive economic supports from your non-coresident other relatives, excluding cash gift?” that takes a value of 1 to an affirmative response, and 0 otherwise. Furthermore, two intersection variables were established.

##### Average Frequency of Meeting With Living Children

First, responses to the question “How often do you meet your child?” are ranked as follows: 1 (almost never), 2 (once a year), 3 (once every 6 months),4 (once every 3 months), 5 (once a month), 6 (every 2 weeks), 7 (once a week), 8 (2-3 times a week), and 9 (almost every day). First, all responses for living children were summed up to measure the total frequency scores of meetings. If no living children exist, a value of 0 is assigned to the variable. Second, the average frequency of meeting with living children is calculated as the total frequency divided by the total number of living children. Higher frequency scores indicate that older people meet their children more frequently. Finally, this variable intersects with main independent variables to measure the moderating effects.

##### Average Frequency of Contact With Living Children

Responses to the question “How often do you have contact with your child either by phone, text message, mail, or email, when you didn't live with him/her?” are rated as follows: 1 (almost never), 2 (once a year), 3 (once every 6 months),4 (once every 3 months), 5 (once a month), 6 (every 2 weeks), 7 (once a week), 8 (2-3 times a week), and 9 (almost every day). Indeed, the total frequency scores were added up and divided by living children to obtain the average frequency of contact with living children. Higher scores represent a higher frequency of contact with living children. The average frequency of contact with living children intersects with main independent variables separately to measure the moderating effects of family support.

#### Control Variables

##### Age

This is a cumulative variable. Age is a moderator between widowhood and senior loneliness (33). Indeed, stress-buffering of social support is more effective toward the oldest-old than the young-old (16).

##### Gender

This is assigned a value of 1 for female individuals, and 0 for male individuals.

##### Marriage

An individual's marital status is a binary variable that takes the value of 1 for married individuals, including married but not living with spouse temporarily and cohabitated, and 0 for separated, divorced, widowed, and never married.

##### Urban

Studies have found that the mental health of Chinese older people in urban and rural areas is different. The scores of depression and anxiety of urban older people are higher than those of rural older people (75). In contrast, mental health level of rural older people is generally lower than that of urban older people (76). Therefore, the study merged individuals who lived in the main city zone, a combination zone between urban and rural areas, and town center. Otherwise, the study merged individuals who live in special areas, township centers, and villages. Therefore, variable of urban is a binary variable with a value of 0 for country living and 1 for urban living.

##### Education

The responses to the highest level of education attained by the individual are ranked at 11 levels: 1 (illiterate), 2 (did not finish primary school), 3 (home school), 4 (elementary school), 5 (middle school), 6 (high school), 7 (vocational school), 8 (2-/3-year college/associate degree), 9 (4-year college/Bachelor's degree), 10 (Master's degree), and 11 (Doctoral degree/Ph.D.).

##### Pension

Having financial difficulties and being unable to pay bills for medicine can deteriorate the mental health of the older people (50). Pension income improves mental wellbeing in the older people and moderates the effects of depression as it helps secure their financial resources (77, 78). Although the basic pension cannot support the daily expenses of most rural elderly people, its spiritual effect on the older people is much higher than its economic effect (61). Therefore, pension is a binary variable that takes 1 if the individual was included in at least one pension program, and 0 otherwise. Pension programs cover pensions and supplemental pensions from the government, institutions, and firms; new cooperative pension programs; urban and rural resident pension programs; land expropriation pension insurance; old age pension allowance; life insurance; commercial pension insurance; and other pension programs.

##### Medical Insurance

Economic factors are an essential factor in access to medical services; indeed, expansion of healthcare insurance coverage has reduced the medical costs of those patients with low socioeconomic status (79). Therefore, this is a binary variable that takes 1 if it includes at least one medical insurance, and 0 otherwise. Medical insurances covered 10 different programs: urban employee medical insurance, urban resident medical insurance, new cooperative medical insurance, urban and rural resident medical insurance, government medical insurance, medical aid, private medical insurance purchased by work unit, private medical insurance purchased by individual, urban non-employed health insurance, and other medical insurance.

## Results

Table 1 presents the descriptive analysis. The mean value of depression among the 7,986 Chinese older adults included in the study was 18.36. In total, 66.5% of older people once experienced at least one stressful life event, and on average, 2.81 stressful life events were experienced. The average social activity was 0.754, which was <1 activity, and 53.4% of the 9619 Chinese older adults did not participate in social activities. In total, 45.2% and 55.2% of respondents received financial support from their relatives and children. The mean of the average frequency of meeting and contact with living children were 2.449 and 1.722, respectively, which was considered relatively infrequently connected. The sample was nearly equally distributed by gender, with 50.4% females and 49.6% males. However, living in urban or rural areas was not distributed equally, with 28.2% of the Chinese older people living in urban areas. A total of 78.9% reported being in the relationships, and 79.6% and 97.7% had pension and medical insurance, respectively.

**Table 1:** Descriptive statistics of the variables.

**Variable**	**Obs**.	**Mean**	**Std.Dev**.	**Range**
Depression	7,986	18.36	6.541	[10,40]
Number of stressful life events experienced	9,619	2.810	1.399	[0,8]
Experienced stressful life events	9,619	0.665	0.472	[0,1]
Social activity	9,619	0.754	1.036	[0,9]
Living children	6,008	3.145	1.673	[0,15]
Financial support by relatives	9,619	0.452	0.498	[0,1]
Financial support by children	9,619	0.552	0.497	[0,1]
Satisfaction with relationship with children	8,712	2.391	0.753	[1,5]
Average frequency of meeting with living children	9,619	2.449	2.760	[0,9]
Average frequency of contact with living children	9,619	1.722	2.475	[0,9]
Age	9,619	68.58	7.076	[60,105]
Gender	9,619	0.504	0.500	[0,1]
Marriage	9,617	0.789	0.408	[0,1]
Education	8,269	2.885	1.879	[1,10]
Urban	9,587	0.282	0.450	[0,1]
Pension	9,547	0.796	0.403	[0,1]
Medical insurance	9,619	0.977	0.151	[0,1]

As shown in Table 2, experiencing more stressful life events significantly increased depression in the Chinese older people. When considering the variable of social activities, although the Chinese older people have experienced cumulative stressful life events, more social activities can decrease the risk of depression. As for variable of family support, the number of children has an inverted- U shape effect on depression. When the older people experienced more stressful life events, financial support from relatives and children can decrease depression. When the average frequency of meeting and contact is higher, older people who have experienced cumulative stressful life events report lower depression. However, if the older people are not satisfied with their children, the negative effects from stressful life events accumulate and significantly increase their depression. As for control variables, cohort with older old, male, living in the urban areas, higher levels of education can decrease risk of depression. As 97.7% of older people owned medical insurance, where medical insurance variable has an extremely skewed distribution, this variable must be omitted in further analyses.

**Table 2:** Results from multiple regression model of risk factors and social support when experienced multiple stressful life events predicting depression.

**Model**	**Stressful life event**	**Social activity**	**Family support**	**Social support**
Number of stressful life events experienced (NE)	1.556***(0.078)	1.727***(0.094)	1.595***(0.427)	1.810***(0.435)
**Moderator variable of social activities**				
Social activities		0.262(0.182)		0.300(0.232)
Social activities with NE		−0.244***(0.060)		−0.225***(0.069)
**Moderator variable of family support**				
Living children			0.527***(0.183)	0.504***(0.182)
Quadratic form of living children			−0.028(0.020)	−0.027(0.020)
Financial support by relatives			1.548(0.966)	1.604*(0.967)
Financial support by children			1.308(1.156)	1.321(1.155)
Satisfaction with relationship with children			1.315***(0.356)	1.380***(0.355)
Average frequency of meeting with living children			0.096(0.106)	0.085(0.105)
Average frequency of contact with living children			0.142(0.105)	0.138(0.104)
Financial support by relatives with NE			−0.363(0.252)	−0.344(0.253)
Financial support by children with NE			−0.305(0.308)	−0.315(0.307)
Satisfaction with relationship with children with NE			0.160(0.098)	0.133(0.098)
Average frequency of meeting with NE			−0.060*(0.033)	−0.056*(0.032)
Average frequency of contact with NE			−0.034(0.032)	−0.031(0.031)
**Control variables**				
Age	−0.048***(0.012)	−0.055***(0.012)	−0.088***(0.016)	−0.094***(0.016)
Gender	1.718***(0.159)	1.757***(0.159)	1.788***(0.207)	1.825***(0.206)
Marriage	0.603**(0.241)	0.512**(0.240)	0.420(0.257)	0.355(0.256)
Education	−0.408***(0.043)	−0.353***(0.044)	−0.379***(0.054)	−0.330***(0.054)
Urban	−1.129***(0.177)	−1.048***(0.177)	−1.006***(0.227)	−0.909***(0.228)
Pension	−0.056(0.187)	−0.015(0.186)	0.050(0.232)	0.082(0.231)
Medical insurance	1.269**(0.504)	1.155**(0.500)	0.629(0.672)	0.612(0.664)
Constant	16.058***(1.098)	16.287***(1.103)	13.262***(1.987)	13.213***(1.996)
Observations	6868	6868	4248	4248
R-squared	0.134	0.141	0.190	0.197

*** p < 0.01, ** p < 0.05, * p < 0.10. Robust standard errors are in parentheses.

Therefore, in order to find covariates to apply PSM analysis, based on multiple regression outcomes, the study selected the variables of age, gender, marital status, educational levels, urban, and pension to measure the factors related to whether the Chinese older people experienced stressful life events by means of logistic regression. Furthermore, variables of total number of social activities in social activity model and the number of children, financial support from relatives and children, satisfaction with children, the average frequency of meeting and contact with children in family support model are selected. The study applied nearest neighbor matching, radius matching, and kernel matching to measure the propensity score of the four models.

In Table 3, one-to-one nearest neighbor matching shows that the stressful life events experienced significantly increased the risk of depression in 2.929. In the model of social activity, after one-to-one matching, the risk of depression increases to 2.982, which presents no clear buffering effects from social activity. The models, including family support and social support, were insignificant. For radius matching, a quarter of the standard deviation of the estimated propensity value of the sample may be used as the caliper size (67). Therefore, the study set 0.01 as the caliper size. Matching similar resources between treated and control groups, the stressful life events experienced significantly increased the risk of depression by 2.969. When engaging in social activity, the risk is enhanced to 2.981. However, the risk of depression significantly decreases to 1.938 and 1.962 when considering models of family support and social support, respectively, which explains the buffering effects of family support. Kernel matching presents the same trend as radius matching but a much stronger buffering effect from family support on depression. The moderating effects from stressful life events on depression on radius matching are 1.031 in the family support model (2.969 vs. 1.938) and 1.007 in the social support model (2.969 vs.1.962); however, the values are 1.150 (2.942 vs. 1.792) and 1.080 (2.942 vs. 1.862), respectively in kernel matching.

**Table 3:** Results from propensity score matching (PSM) and bias-corrected matching from stressful life events and social support predicting depression.

**Model**	**Stressful life event**	**Social activity**	**Family support**	**Social support**
One-to-one nearest neighbor matching	2.929***(0.480)	2.982***(0.503)	1.466(1.050)	1.271(1.108)
Radius matching with caliper size 0.01	2.969***(0.348)	2.981***(0.344)	1.938**(0.944)	1.962**(0.939)
Kernel matching	2.942***(0.347)	2.956***(0.344)	1.792**(0.887)	1.862**(0.894)
Bias-corrected matching	3.029***(0.368)	3.049***(0.361)	2.021***(0.260)	1.932***(0.245)

*** p < 0.01, ** p < 0.05, * p < 0.10. Standard errors are in parentheses.

Furthermore, in bias-corrected matching, stressful life events experienced significantly increased the risk of depression in 3.029; however, it decreased to 2.021 with the help of family support. Indeed, estimators of bias-corrected matching strengthen social activity buffering effects, which decrease the effects of experienced stressful life events on depression to 1.932 under the condition of social support.

To check match quality, the study conducted a data balancing test. The results are listed in Table 4. Based on the significance of the models, the study selected radius matching and kernel matching to make comparisons for better application of PSM. The pseudo R-square decreased for both matches of the model of stressful life event. The mean bias decreased strongly in radius matching from 26.2% to 5.1%. In the social activity model, the pseudo R-square and mean bias decreased significantly from 0.144 to 0.008 and from 22.6 to 5.3%, respectively, in radius matching. It shows the same trend in the family support model and social support model, which indicates that radius matching is a much more effective method. Therefore, in the robustness check, the study uses the outcomes of radius matching to further check the models' robustness and shrink the sample to the matched ones.

**Table 4:** Result comparisons of data balancing test under the method of radius and kernel matching in propensity score matching (PSM).

**Method**	**Radius matching**			**Kernel matching**		
**Sample**	***Pseudo R* ^2^**	**LR chi2**	**Mean bias**	***Pseudo R* ^2^**	**LR chi2**	**Mean bias**
**Model of stressful life event**						
Unmatched	0.144	1266.7	26.2	0.144	1266.7	26.2
Matched	0.008	88.94	5.1	0.008	90.91	5.4
**Model of social activity**						
Unmatched	0.144	1267.98	22.6	0.144	1267.98	22.6
Matched	0.008	93.01	5.3	0.009	97.58	5.9
**Model of family support**						
Unmatched	0.182	750.46	27.3	0.182	750.46	27.3
Matched	0.078	667.45	18.7	0.079	683.75	18.8
**Model of social support**						
Unmatched	0.182	751.10	25.7	0.182	751.10	25.7
Matched	0.083	709.03	18.6	0.084	722.64	18.7

In the model of robustness check (Table 5), whether the Chinese older people experienced stressful life events was selected as the main independent variable, which presents positive effects on depression. Social activities can buffer the effects of stressful life events and depression in the negative direction of the intersection. As for variables of family support, children's contribution still has an inverted-U shape effect on depression. Indeed, greater dissatisfaction with children increases depression levels in the older people. Furthermore, more frequent meeting and contact with children can moderate the effects of stressful life events on depression. However, considering whether experienced stressful life events affect depression, variables of financial support by relatives and children do not contribute to the effects on depression as effectively as the relationship between the total number of stressful life events experienced and depression. In this qualitative model, variable of financial support by children has negative effects on depression, but the intersection variable between stressful life events and financial support by children does not present similar effects.

**Table 5:** Results from multiple regression model of risk factors and social support when experienced stressful life events after propensity score matching (PSM) analysis predicting depression (Robustness check).

**Model**	**Stressful life event**	**Social activity**	**Family support**	**Social support**
Experienced stressful life events (QE)	3.342***(0.156)	3.714***(0.195)	1.091(1.276)	1.549(1.291)
**Moderator variable of social activities**				
Social activities		−0.199**(0.094)		−0.085(0.145)
Social activities with QE		−0.443***(0.126)		−0.457***(0.168)
**Moderator variable of family support**				
Living children			0.654***(0.202)	0.616***(0.202)
Quadratic form of living children			−0.040*(0.023)	−0.039*(0.023)
Financial support by relatives			0.142(0.770)	0.200(0.766)
Financial support by children			−0.166(0.894)	−0.181(0.887)
Satisfaction with relationship with children			1.146***(0.237)	1.136***(0.237)
Average frequency of meeting with living children			−0.007(0.061)	−0.008(0.061)
Average frequency of contact with living children			0.061(0.060)	0.070(0.060)
Financial support by relatives with QE			0.098(0.854)	0.135(0.851)
Financial support by children with QE			0.219(1.013)	0.230(1.006)
Satisfaction with relationship with children with QE			0.831***(0.284)	0.791***(0.283)
Average frequency of meeting with QE			−0.096(0.076)	−0.084(0.076)
Average frequency of contact with QE			−0.040(0.074)	−0.039(0.073)
**Control variables**				
Age	−0.027*(0.014)	−0.034**(0.014)	−0.075***(0.018)	−0.080***(0.018)
Gender	1.858***(0.164)	1.899***(0.164)	1.876***(0.217)	1.915***(0.217)
Marriage	−0.175(0.267)	−0.208(0.268)	−0.108(0.267)	−0.140(0.268)
Education	−0.442***(0.044)	−0.378***(0.045)	−0.427***(0.055)	−0.372***(0.056)
Urban	−1.192***(0.182)	−1.106***(0.182)	−1.059***(0.234)	−0.957***(0.236)
Pension	−0.054(0.195)	−0.001(0.194)	0.104(0.244)	0.155(0.244)
Medical insurance	1.477***(0.553)	1.264**(0.548)	0.454(0.799)	0.410(0.796)
Constant	17.562***(1.153)	18.140***(1.151)	17.408***(1.846)	17.737***(1.848)
Observations	6426	6416	3903	3887
R-squared	0.128	0.135	0.161	0.166

*** p < 0.01, ** p < 0.05, * p < 0.10. Robust standard errors are in parentheses.

## Conclusions and Discussions

Chinese older people who experience stressful life events are likely to experience an increase in their depression, which is supported by both linear multiple regression analysis and propensity matching analysis. Hypothesis 1 is thus supported.

Stressful life events, especially bereavement, leading to depression in Chinese older people are affected by Chinese cultural contexts. As a Confucian country, China is a family-concentrated society, which is tightly related to the bonds of kin and marriage. Social networks of older people decrease as age increases (33, 34); consequently, emotional closeness with family members is stronger. Besides, due to the stigmatization of mental disorders, the negative stereotypes by the inadequate understanding of depression from community and individual, older people may be hesitant to express their feelings and refuse to receive professional treatments of symptoms, when they experienced stressful life events.

In detail, first, bereavement refers to the losses of close network partners, which gives rise to short-term negative emotional shocks and even long-term psychological trauma; for example, widowhood implies that older people have lost stable emotional support and resources of functional support (31). Indeed, Chinese inter-generational connections between parents and children are close, and children have become a factor affecting parents' self-efficacy and happiness. Adult children are the main family caregivers toward Chinese older people to supply monetary, instrumental and emotional support under the traditional perspective (59). Therefore, bereavement of children may give rise to destructive effects on older people. Second, most studies have certified high correlation between physical health and mental health (27, 28). For example, functional impairment may lead to depression, and conversely, depression may result in functional impairment. Third, due to decline of cognitive ability, older people are likely to be frauded online (80) and being frauded has made older people feel frustrated, experience financial losses and lose their trust in society, consequently leading to decline of life quality.

Social activity, as a moderator between stressful life events and depression, is supported by linear regression; however, it is not clearly measured by propensity matching analysis, which is partly supported by Hypothesis 2. Under Chinese cultural contexts, Chinese prefer to actively participate in social activities and frequently connect with their neighbors and partners in the community. Furthermore, older people perceive the boundaries of time and prefer to select emotionally meaningful activities through the process of maximizing their experiences of positive emotions and avoiding negative emotions (15, 52). Therefore, social activities give older people an efficient way to relieve the pressure and acquire network support from partners in the community. However, it is essential to consider reverse causality problems, in that health problems and increasing age may prevent the participation of activities (7). Indeed, social activity effects will reverse from positive to negative in very late life (72). In this study, nearly 53.4% of the 9,619 respondents did not participate in social activities.

When the respondents experienced stressful life events, family support significantly buffered its negative effects on depression, which supports Hypothesis 3.

When older people experienced stressful life events, with the perception of limited time, they instinctively value close social ties and loved ones as main supporters. Emotional support in the family alleviates older people's shocks from stressful life events and helps older people remain positive psychological condition. An adaptation cycle toward trauma exposure is described as follows: in the early phase, grief rises and individuals tend to withdraw social relations; as the grief loses over time, individuals are likely to resume their life and resolve what has happened (14, 34). Therefore, emotional support suited with adaptation cycle presents the following status: first, family support as an effective way has helped older people decrease their intensity of grief in the early phase of exposure to stressful life events; second, duration of grief will be consequently shortened by family support. Economic support, as a form of family support, helps older people secure their financial resources to keep stable life. For example, depression outpatient treatment and psychotherapy are out of the scope of medical insurance in most cities (81, 82). With the economic support, old people are likely to lessen their financial burdens and be willing to have treatments. Therefore, when older people, as recipients, perceive the received social support as being sufficient to meet their emotional needs, their efficacy, confidence, and self-esteem are enhanced, which consequently increases their appraisal of their own ability to deal with stress. Indeed, close contact and meeting between older people and their adult children decrease the risk of older people being financially frauded.

As for Hypothesis 4, the effects of social support, as a moderator between stressful life events and depression, are supported by linear regression analysis and propensity matching of bias-corrected estimators. Under social support, which covers family support and social activities as a whole, the buffering effects of stressful life events on depression are more decreasing, which also reflects the effects of social activities. Social activity and family support are guided by policies and specific measurements to take effects. For one thing, family policy encourages Chinese to carry forward the traditional virtues of supporting the older people to strengthen inter-generational close relationship. For another, policies and specific measures support the construction of the platforms to facilitate social activities of older people, such as the universities for the aged, senior care centers, and sports parks equipped with smart fitness facilities. Currently, policies mainly focus on public service issues such as activities and care toward Chinese older people. However, policies and specific measurements are not sufficient toward depression in Chinese older people; and services for the prevention and treatment of mental disorders are inadequate either.

Therefore, the negative effects of stressful life events on depression can be partly erased, when older people perceive support from family and social activities. Indeed, the negative effects will be minimal, if older people receive sufficient family support and take part in diverse social activities, such as complete satisfaction with relationship with children, daily or weekly contact and meeting with children.

The intervention for and prevention of mental disorders in Chinese older people are based on four aspects. First, Chinese still regard kinship and filial piety as an essential value in life. Family members should consciously pay attention to older people's mental condition and provide effective support that meets their needs, especially when older people experienced stressful life events. Second, older people and their neighbors and community partners mutually support each other in the close partnership. When older people experience stressful life events, network partners should frequently communicate with older people and encourage them to social activities. Third, public policy should support community health service centers financially and professionally to make it capable of mental health services. For example, early detection of mental disorders by means of incorporating emotional assessment into the daily physical examination of older people; continuous attention to risky groups; and psychotherapy to the patients. Fourth, the public should improve awareness of mental disorders by publicizing the knowledge of depression through community lectures, bulletin boards and other various ways to erase its stigmatization, enlarge the public covers of medical insurance, and encourage business medical insurance to supply diverse plans to help mitigate patients' burdens.

Due to interviewees' recall accuracy and different willingness to disclose, this study has certain limitations. First, the seniors retrospectively self-reported the stressful life events may lead to recall bias. Second, the study makes use of the 2015 CHARLS panel data to discuss the effects of the previously experienced stressful life events on depression and downplays the limits of time when stressful life events occurred. Although bereavement effects on depression have time differences between the short and long terms (33), bereavement also causes life-course pain (51). Therefore, the bereavement of spouse and children, financial problems, and some health adversities throughout the lifetime are discussed in this study. Third, the selected stressful life events have been regarded as a whole in the study, and each event has been assumed to have same psychological impacts on the interviewee, both of which lack event heterogeneity. For example, the bereavement, by an accident or peaceful death after suffering severe illness over several years, can bring about great differences in the traumatic effects on individuals. To eliminate recall bias, future research can make use of a third party to recall the stressful life event that the individual once experienced and measure individuals' depressive feelings and behaviors. Differentiating stressful life events can help measure individuals' responses in greater detail.

## Data Availability Statement

Publicly available datasets were analyzed in this study. This data can be found here: China Health and Retirement Longitudinal Study (CHARLS), http://charls.pku.edu.cn/en.

## Author Contributions

XY and SL: Conceptualization and methodology. SL: Writing—original draft preparation. XY: writing—review and editing. All authors have read and agreed to the published version of the manuscript.

## Conflict of Interest

The authors declare that the research was conducted in the absence of any commercial or financial relationships that could be construed as a potential conflict of interest.

## Publisher's Note

All claims expressed in this article are solely those of the authors and do not necessarily represent those of their affiliated organizations, or those of the publisher, the editors and the reviewers. Any product that may be evaluated in this article, or claim that may be made by its manufacturer, is not guaranteed or endorsed by the publisher.
